# Routes to probe Bismuth induced strong-coupling superconductivity in bimetallic BiIn alloys

**DOI:** 10.1038/s41598-017-09831-9

**Published:** 2017-08-25

**Authors:** Ashish Chhaganlal Gandhi, Sheng Yun Wu

**Affiliations:** Department of Physics, National Dong Hwa University, Hualien, 97401 Taiwan

## Abstract

We report the observation of strong electron-phonon coupling in intergranular linked BiIn superconductors over an infinite range mediated by low-lying phonons. An enhanced superconducting transition temperature was observed from the magnetization, revealing a main diamagnetic Meissner state below T_C_(0) = 5.86(1) K and a critical field H_C_(0) = 1355(15) Oe with an In_2_Bi phase of the composite sample. The electron-phonon coupling to low lying phonons is found to be the leading mechanism for observed strong-coupling superconductivity in the BiIn system. Our findings suggest that In_2_Bi is in the strong-coupling region with T_C_(0) = 5.62(1) K, λ_ep_ = 1.45, ω_ln_ = 45.92 K and *α* = 2.23. The estimated upper critical field can be well-described by a power law with *α* value higher than 2, consistent with the strong electron-phonon coupling.

## Introduction

Recently a number of electron-phonon coupled bi- and tri-metallic superconductors with enhanced superconducting transition have been discovered, renewing interest in conventional phonon-mediated superconductivity^1–4^. We consider the BiIn bimetallic system, in which Bi is a semimetal and In is a weak-coupled superconductor with transition temperature T_C_(0) = 3.4 K (at ambient atmosphere). When annealed together they form In_2_Bi, In_5_Bi_3_ and InBi bimetallic compounds and a solid solution, *α*-In^5^. The reported T_C_(0) for single crystals of In_2_Bi and In_5_Bi_3_ are 5.1 K and 4.1 K, respectively, whereas InBi is a non-superconductor down to 0.5 K. Hutcherson *et al*.^6^ reported the two-gap superconductivity in a composite (In_2_Bi + In_5_Bi_3_) superconductor with T_C1_(0) = 5.6 K and T_C2_(0) = 4.1 K. A microscopic examination of polished InBi samples revealed a heterogeneous mixture of crystal grains, except for pure crystalline phases indicating granular superconductivity. In such a system, the Josephson tunneling between superconducting grains establishes inter-granular links and results in macroscopic superconductivity^7^. However, a comprehensive study, including electron-phonon coupling strength and the enhanced T_C_ in the InBi system, has not yet been carried out. Conventionally, BCS-Eliashberg theory showed that T_C_ is the combined effect of electron-phonon coupling strength (*λ*_*ep*_) and phonon energy (*ω*_*ln*_). In this study, we present a transition of weak to strong electron-coupling superconductivity by tuning the Bi concentration in bimetallic Bi_y_In_1-y_ alloys, consisting of two superconducting gaps at y = 0.1, 0.2, 0.6, and 0.7 and a single gap at y = 0.01, 0.2, 0.3, 0.4 and 0.5, respectively. The enhancement of T_C_(0) = 5.62(1) K was observed at y = 0.3, led by low lying phonons that can be well described by using Allen and Dynes’ theory^2^.

## Results

### Crystal structure analysis of BiIn alloy

**Figure 1:**
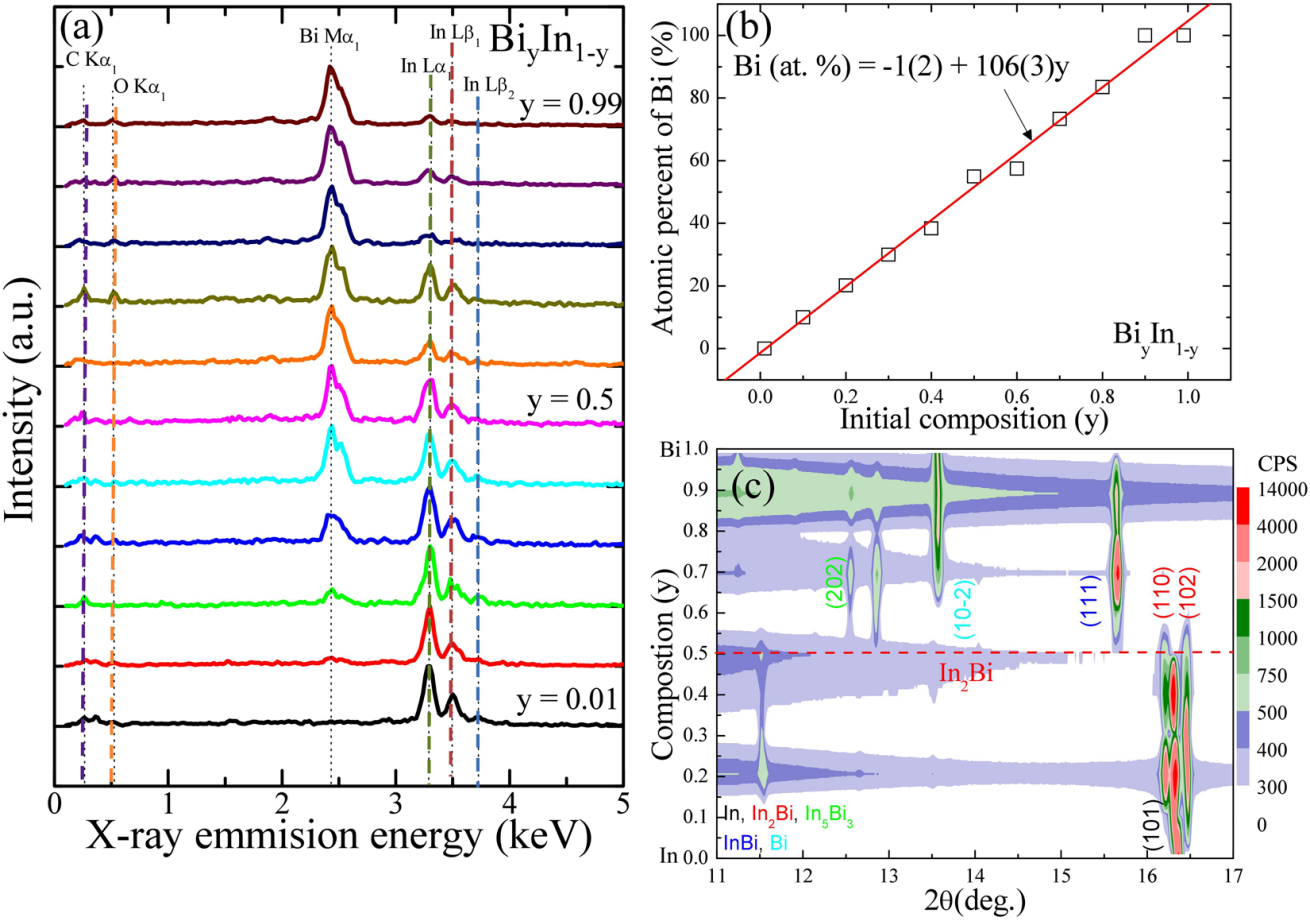
The EDS and x-ray analysis. (**a**) Typical EDS elemental spectra, revealing a series of peaks associated with elemental Bi and In, verifying that all samples contain only Bi and In elements. (**b**) Plot of bismuth atomic percentage verses initial composition y, where the solid line is a linear fit to the data points. (**c)** A 2D plot of XRD patterns of Bi_y_In_1-y_ bimetallic alloys over a narrow 2*θ* scattering range.

**Figure 2:**
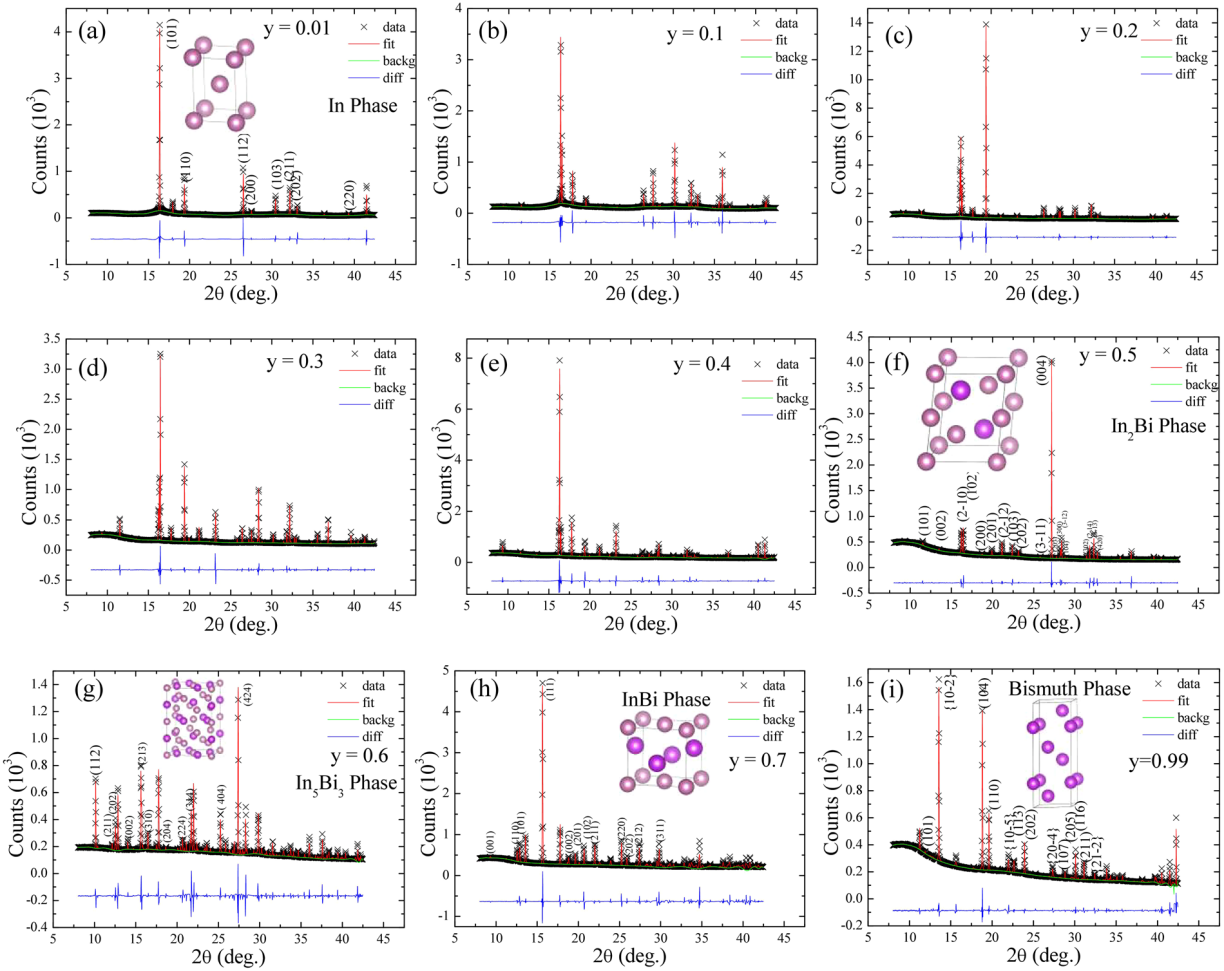
The crystal structure and x-ray refinements analysis. (**a**–**g**) The observed and Rietveld refined (gray solid line) X-ray diffraction patterns of Bi_y_In_1-y_ bimetallic alloys.

Figure 1(a) presents the typical EDS spectra, showing a series of elemental Bi and In constituents that can be assigned to Bi-Ma_1_, In-Lα_1_, In-Lβ_1_, and In-Lβ_2_, respectively. The small peaks of C and Cu were the result of the carbon film on the Cu grid from mounting the sample. Moreover, the atomic percentage of Bi increases linearly with a slope of 106(3) Bi at. %/y and approaches the value of bulk Bi (y > 0.9), as shown in Fig. 1(b). The observed atomic % Bi is in good agreement with initial composition y. The structural analysis was conducted by room temperature X-ray diffraction (XRD) using synchrotron radiation (BL-01C2) at the National Synchrotron Radiation Research Center in Taiwan, with an incident wavelength of λ = 0.7749 Å. For detailed investigation of the crystalline phase of bimetallic alloys, the SR-XRD technique must be employed, as this is otherwise quite difficult using the usual XRD techniques. A 2D plot of the XRD pattern of Bi_y_In_1-y_ (0.01 ≤ y ≤ 0.99) bimetallic alloys over a narrow scattering range of 2*θ* is as shown in Fig. 1(c), where the vertical axis represents the starting composition y. Different colors were used to differentiate the peak intensities of the diffraction pattern. From the above 2D plot, for y = 0.01 a single nuclear peak (101) indexed based on *I4/mmm* (No. 139) becomes visible, indicating the formation of an In-solid solution having the same structure as that of virgin In. Since the atomic radii of Bi (156 pm) is smaller than In (167 pm), Bi can therefore occupy interstitial sites in the In lattice and can form an interstitial solid solution^8^. The In phase becomes unstable beyond y = 0.1 and shows the presence of two immiscible phases in each alloy up to y = 0.4. A horizontal red-colored dashed line drawn at y = 0.5 marks the two nuclear peaks (110) and (102) indexed based on *P6*_3_ */mmc* (No. 194), indicating the formation of a pure In_2_Bi crystalline phase. For y = 0.6, two additional nuclear peaks (202) and (111) become visible indexed based on *I4/mcm* (No. 140) and *P4/nmm* (No. 129), respectively indicating the formation of immiscible In_5_Bi_3_ and InBi crystalline phases. For y from 0.7 to 0.99, along with InBi one additional nuclear peak (10–2) becomes visible indexed based on *R-3m* (No. 166), and trigonal-hexagonal scalenohedral Bi indicates the presence of two immiscible phases in each alloy. In bimetallic alloys, because of the different atomic and structural properties of the constituent’s elements and the state of the alloy (pure or mixed), lattice expansion or contraction is unavoidable. The detailed structural analysis of the x-ray diffraction pattern of Bi_y_In_1-y_ bimetallic alloys was further refined by Rietveld analysis^9^ using the GSAS software package^10^. The refined patterns and the fitting parameters are as shown in Fig. 2. From the refined XRD spectra of Bi_y_In_1-y_ bimetallic alloys, it was observed that depending on their initial composition, formed alloys are either in pure or mixed form. A single phase was obtained at y = 0.01, as shown in Fig. 2(a), exhibiting the expected In (S) solid solution structure. Since the atomic radii of Bi is smaller than In, Bi can therefore occupy interstitial sites in the In lattice and form an interstitial solid solution. The major detectable phase was In (S), along with In_2_Bi (S) as a secondary phase, for the region of y = 0.1 to 0.4, presenting two immiscible phases in this concentration region, as shown in Fig. 2(b–e). After this stage, the reactant phases have converted fully into In_2_Bi (S) for x = 0.5, as shown in Fig. 2(f). It is to be noted that there is a retardation in the growth of In_2_Bi (S) as the percentage of Bi increases. Structural transformation was observed as y is larger than 0.5, and then a new immiscible phase including In_5_Bi_3_ (S) and InBi (N) was detected at y = 0.6, forming a co-existence of a superconductor (S) and a normal (N) bimetallic compound, and the pairing correlation between electrons is delivered from the superconducting phase to the normal phase through proximity effect (PE), as shown in Fig. 2(g). Figure S1(a) represents the observed state of the alloy (i.e. pure or mixed phases) with respect to initial composition y and the corresponding crystal structure of In, In_2_Bi, In_5_Bi_3_, InBi and Bi, respectively (see the supporting information: Figure S1). The formed pure In_2_Bi phase at y = 0.5, is marked by a red circle. The observed newly formed compounds with respect to the initial composition contradict with previous finding. Giessen *et al*.^11^ reported the equilibrium phase diagram of In-Bi according to which pure In_2_Bi, In_5_Bi_3_ and InBi are formed at 33.5(0.5), 37.5(5) and 50 atomic % of Bi. The reason for the observed discrepancy is not clear yet, but for our alloys the estimated atomic % and structural properties are in good agreement with the initial In-Bi composition. The effect of Bi doping in In (solid solution) and the effect of strain on the lattice constants of the In-solid solution, In_2_Bi, In_5_Bi_3_ and InBi, is plotted with respect to initial composition y, as shown in Figure S1(b–e **)** (the dotted line represents the standard values^5^). Lattice expansion was observed for the In-solid solution, InBi and Bi phases, and contraction was observed for the In_5_Bi_3_ phase of the mixed phase alloys. For In_2_Bi, phase lattice contraction was observed along the axial plane, and expansion along the basal plane. The observed lattice expansion for the solid solution is the combined effects of electron doping, as Bi has one extra electron compared to In and the strain. On the other hand, the observed lattice expansion/contraction in immiscible mixed alloys could be due to strain effect, since thermal coefficients vary from material to material. The atomic position and structural properties of the parent elements, newly formed In_2_Bi (y = 0.5), In_5_Bi_3_ (y = 0.6), and InBi (y = 0.6) crystalline phases obtained after refinement are as tabulated in the supplementary information of Table S1, along with the atomic radii of In and Bi. The observed lattice constants of the different crystalline phases are in excellent agreement with previously published results. As tabulated in Table S1, it can be observed that the *c/a* of In_2_Bi, In_5_Bi_3_ and InBi, 1.1992, 1.4830 and 0.9548, respectively, is smaller than that of both In and Bi, 1.5208 and 2.6025, respectively. Such a contracted lattice, compared to that of the constituent elements, may result in softening of the vibrational mode, that is, phonon softening, thus giving rise to the enhanced electron-phonon coupling strength. However, it has been reported that In_2_Bi and In_5_Bi_3_ are superconductors below 5.1 and 4.1 K, respectively, whereas InBi is non-superconducting down to 0.5 K^6^. Allen *et al*.^2^ observed strong-coupling behavior for the In_2_Bi phase mediated by low energy phonons. Therefore, studying the superconducting properties of In-Bi bimetallic alloys will provide more insight into these newly formed compounds.

### Temperature Dependency of ZFC and FC Magnetization

**Figure 3:**
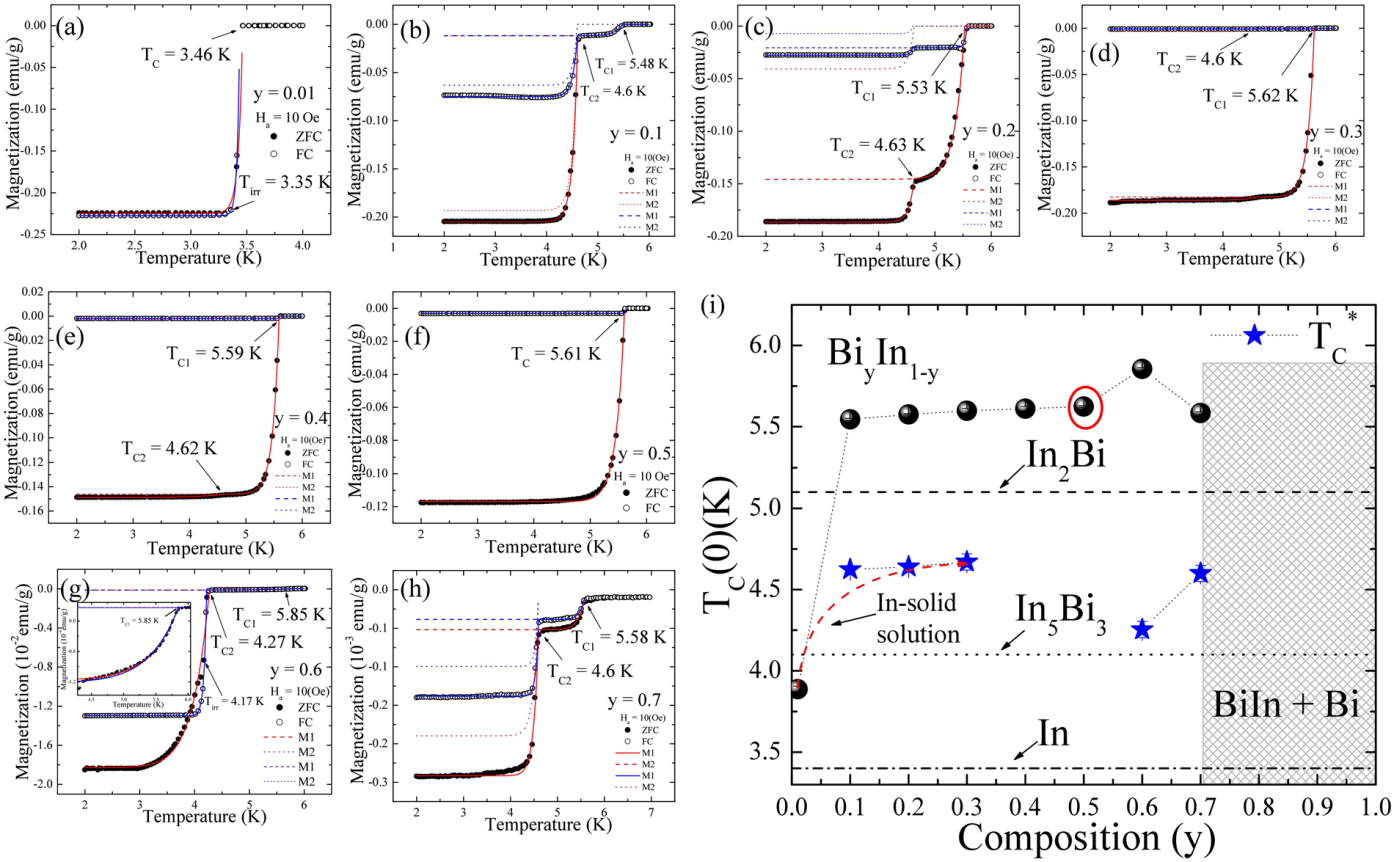
The temperature dependence of M(T). (**a**–**h**) Temperature dependency of magnetization measured between 2 to 6 K in an external magnetic field of H_*a*_ = 10 Oe in the ZFC (shielding) and FC (Meissner) modes. The red and blue solid curve presents the fitted curve of the modified London expression to the data. (**i**) Fitted value of transition temperature T_C1_ (black sphere) and T_C2_ (blue star) in H_*a*_ = 10 Oe as a function of initial composition.

A superconducting material when cooled below unique superconducting transition temperature T_C_ shows perfect Meissner effect. However in Bi_y_In_1-y_ bimetallic alloys, the effect of electron doping, strain effect, and superconducting proximity (SPE)^12^ structural and vibrational properties of newly formed In_2_Bi, In_5_Bi_3_ and InBi compounds on the superconducting properties are unavoidable. To study the superconducting properties of these bimetallic alloys we carried out detailed field and temperature dependent magnetization measurements. Magnetic properties were measured using a Quantum Design MPMS VSM SQUID magnetometer. The transition temperature below which the diamagnetic Meissner effect becomes dominant was confirmed by measuring the zero-field cooled (ZFC) and field-cooled (FC) magnetization. The ZFC is a screening measurement, performed by first cooling the sample in a zero magnetic field to 2 K, subsequently applying a small magnetic field, H_*a*_ = 10 Oe, and then measuring the resultant magnetization while warming the sample. The FC is the Meissner flux expulsion measurement, performed by first cooling the sample to 2 K in a small magnetic field, H_*a*_ = 10 Oe, and then measuring the resultant magnetization while warming the sample. Four selected samples of field cooled and zero-field cooled M(T) measurements recorded in the 1.8–6 K range with H_*a*_ = 10 Oe, as represented in Fig. 3(a–d). As expected, two step-like transitions were observed in Fig. 3(b) due to two immiscible phases that can be described using a superposition of the London equation^13^ for In_2_Bi(M1) and α-In(M2), showing two step-like transitions, T_C1_ and T_C2_, which are consistent with the multiphase bismuth-indium system obtained by Hutcherson *et al*.^6^ and Currie *et al*.^14^. The step-like magnetization can be well fitted using a modified London equation:

1 $${\rm{M}}({\rm{T}})=a+{H}_{a}\times \frac{-1}{4\pi }\{\frac{-3}{2\rho }[1-6(\frac{\lambda }{ < d > })coth(\frac{ < d > }{2\lambda })+12{(\frac{\lambda }{ < d > })}^{2}]\},$$

**Figure 4:**
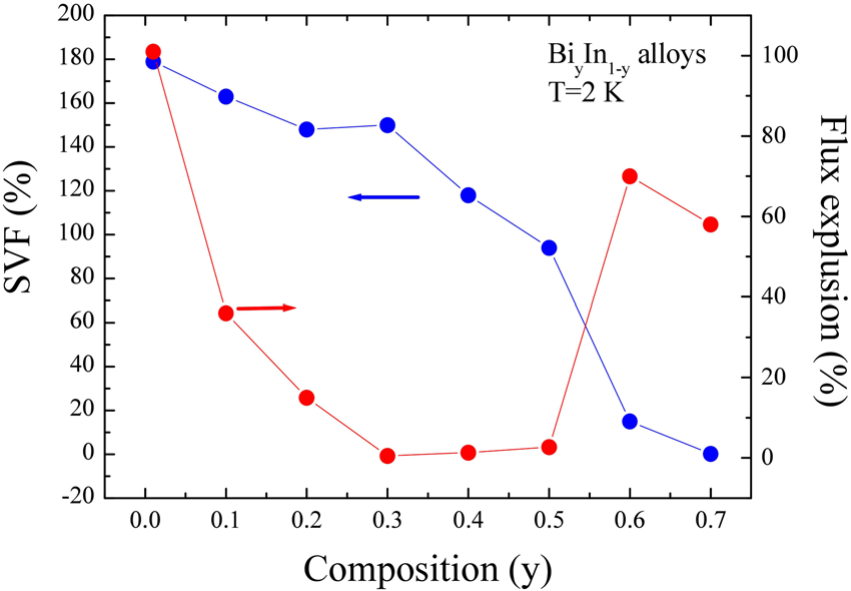
The SVF and flux expulsion analysis. Plot of the calculated values of SVF and flux expulsion with respect to initial composition.

Where <d> is the mean size, *ρ* is mass density, and *λ*_*L*_ is the London penetration depth which varies according to ${\lambda }_{L}(T)={\lambda }_{L}(0){[1-{(T/{T}_{C})}^{P}]}^{-1/2}$. Conventionally, *P* = 4 has been utilized to define bulk-like superconductivity for spherical particles. Li *et al*.^15^ used the above London equation to define the susceptibility in well-separated spherical particles of lead with fix *P* = 4. However, the value of *P* defines only the distribution of the transition temperature, i.e., the higher the value of *P* ( ≥ 1) the steeper the distribution of transition temperature will be. In such a scenario, the bulk-like magnetization M(T), can be well fitted using *P* > 4. The solid red and blue lines shown in Fig. 3(a–h) represent the fitted curves for ZFC and FC, respectively, with M(T) = M_1_ + M_2_, and the steepness of M(T) represented by the value of *P* and the penetration depth at H_*a*_ = 10 Oe. The fitted values of T_C1_(10 Oe) and T_C2_(10 Oe) with respect to the initial composition y are as shown in Fig. 3(i). The highest value of T_C1_ = 5.85 K was observed for the y = 0.6 sample (and the T_C1_ of y = 0.7 is close to that of the In_2_Bi phase), indicating the presence of an In_2_Bi phase. However, from the XRD data (see Fig. 2(g and h)), we did not observe any sign for In_2_Bi phase, which could be due to the relatively low superconducting volume fraction (SVF) of the In_2_Bi phase, as can be seen from the M(T) data. Therefore, the above finding shows the potentiality of superconducting measurement even over synchrotron radiation XRD, particularly for superconducting materials. Apparently, enhanced T_C_ was observed for all superconducting phases. The observed enhanced T_C_ for In_2_Bi and In_5_Bi_3_, as compared to that of the reported value of the respective single crystal, is because of the stress induced strain effect, as can be seen from the refined SR-XRD data. Hutcherson *et al*.^6^ carried out a microscopic examination of BiIn eutectic polished samples and revealed a heterogeneous mixture of crystalline grains of In_2_Bi and In_5_Bi_3_ phases. The reported transitions from a eutectic sample obtained at 35 atomic % of Bi concentration are 5.6 K and 4.1 K for In_2_Bi and In_5_Bi_3_ phases, respectively. These reported values are somewhat lower, but still in good agreement with our finding. However, for higher concentrations, i.e. 40 to 50 atomic % of Bi, only transition temperature, 4.1 K, corresponding to In_5_Bi_3_ has been reported which contradicts our observations. Furthermore, the observed enhanced T_C_ for an In-Bi solid solution, α-In could be the combined effect of enhanced density of state on the Fermi surface and the stress induced strain effect. Since, T_C_ is proportional to density of state and Bi has two extra valence electrons compared to In, this results in an increase of density of state N(E_F_) on the Fermi surface with an increase of Bi concentration. The effect of electron and hole doping has been studied in a Tl-Pb-Bi system (which retains the same *fcc*-Pb structure for 0.8 Tl to 0.2 Bi concentrations) in which Tl has one fewer, and Bi has one more, valence electron than Pb. It has been reported that an increase of N(E_F_) from Tl to Bi resulted in strong electron-phonon coupling mediated by low lying phonon and showed an enhancement of T_C_ from 2.4 to 8 K^16^. However, the effect of stress induced strain on T_C_ cannot be neglected, as samples from y = 0.1 to 0.4 are composite in nature. A normal to superconducting transition, namely T_C1_, was observed for the y = 0.6 sample due to Josephson coupled with an anisotropic superconducting granular system via inter-granular weak links^7^, as shown in Fig. 3(g). The weak link could be achieved through a normal phase (e.g. InBi and Bi) or a superconductor with low T_C_ or the grain boundaries. The calculated superconducting volume fraction (SVF) from the ZFC of the pure In_2_Bi phase (Fig. 3(f)) is 94%, close to the perfect diamagnetism (−1/4π, according to the CGS system), indicating bulk like superconductivity without considering the demagnetization factor. The calculated low value of flux expulsion (from FC) of 2.7%, indicated strong flux pinning, possibly on the grain boundaries of In_2_Bi. The values of SVF and flux expulsion at 2 K with respect to initial composition are as shown in the inset of Fig. 4. The high value of flux expulsion and relatively low value of SVF in superconducting-normal phase composites, y = 0.6 and 0.7, suggest relatively strong-linked grains in these samples. The SVF exceeding 100% in composite y = 0.4 to 0.1 samples is attributed to the magnetization contribution from the two superconducting phases and the demagnetization factor of the sample. Due to the high density of defects and strong flux pinning, a very low value of flux expulsion was observed for these composites. As can be seen from Fig. 3(a), the FC and ZFC curve of y = 0.01, α-In, overlaps below T_C_(10 Oe) = 3.46 K down to lowest available temperature, which reflects reversible behavior due to weaker pinning and/or a lower density of defects. The calculated high value of SVF, 179% is attributed to the demagnetization effect of the sample. As expected, 101% of flux expulsion indicated an ideal, pinning–free bulk sample, with the sample always remaining in thermodynamic equilibrium. The similar reversible region was also observed for the In_2_Bi phase of y = 0.1 and y = 0.6 samples, suggesting that both grain coupling and intra-grain pinning are relatively poor. As we can see, the magnetization SVF for the In_2_Bi phase in the former and later samples is only ~9% and ~0.09%, respectively. Furthermore, the reversibility in the y = 0.6 composite could be due to strain caused by normal vortex cores embedded in the superconducting phase. According to Kogan *et al*.^17^ the strains in this type of composite system arise due to the difference in density of the normal (i.e. InBi) and superconducting (i.e. In_2_Bi and In_5_Bi_3_) phases, which are related to the stress dependency of the critical temperature.

### Isothermal Magnetization and theoretical analysis

**Figure 5:**
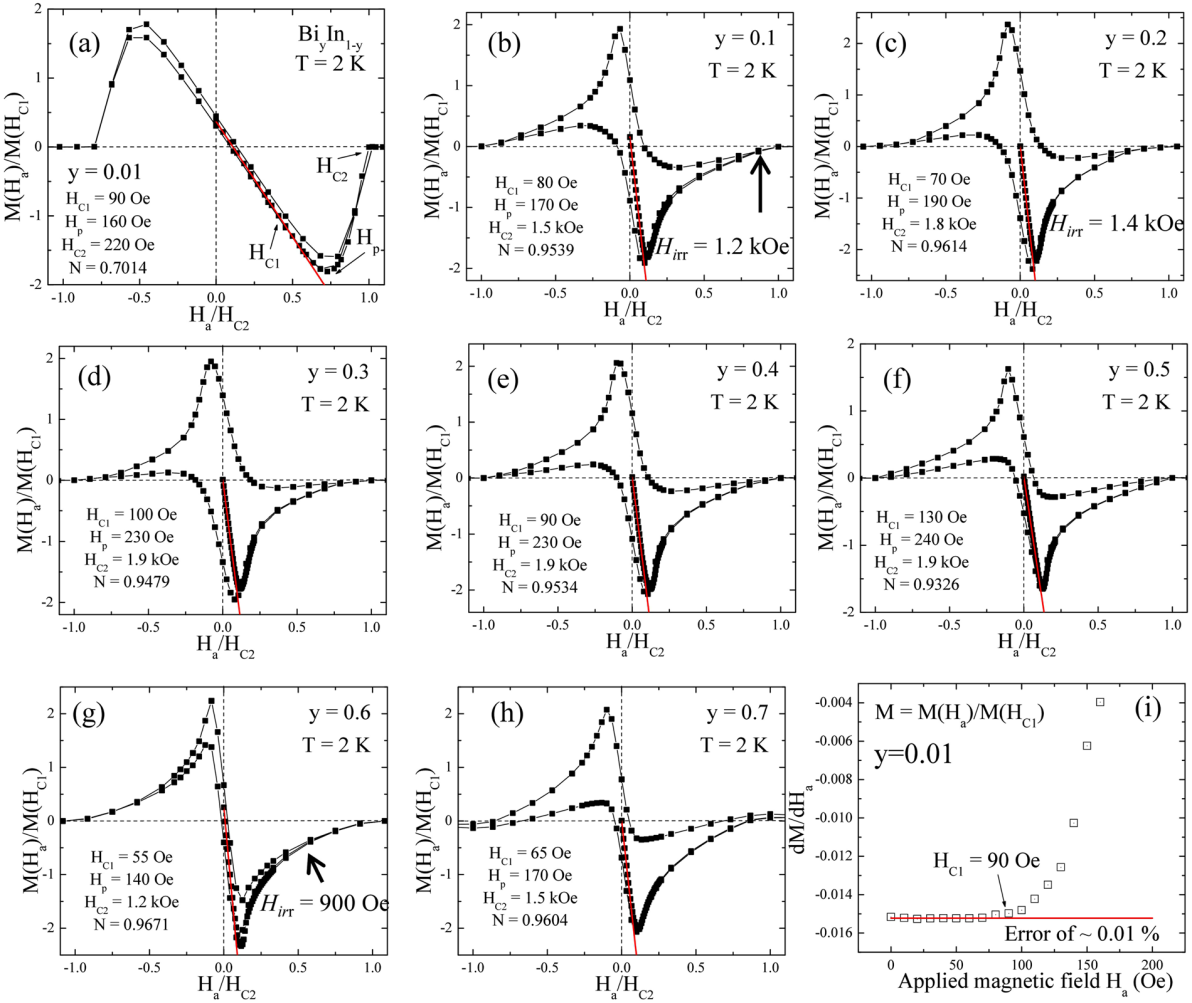
The applied magnetic field dependence of M(H_*a*_). (**a**–**h**) Isothermal magnetization measured at 2 K where the reduced magnetization is defined as the ratio of magnetization M(H_*a*_) to the absolute value at the first critical field M(H_C1_). The red solid line is the linear fit to the first magnetization curve. The observed irreversible field is marked by arrows. (**i**) represents the differential of the first magnetization curve and the linear fit to it.

**Figure 6:**
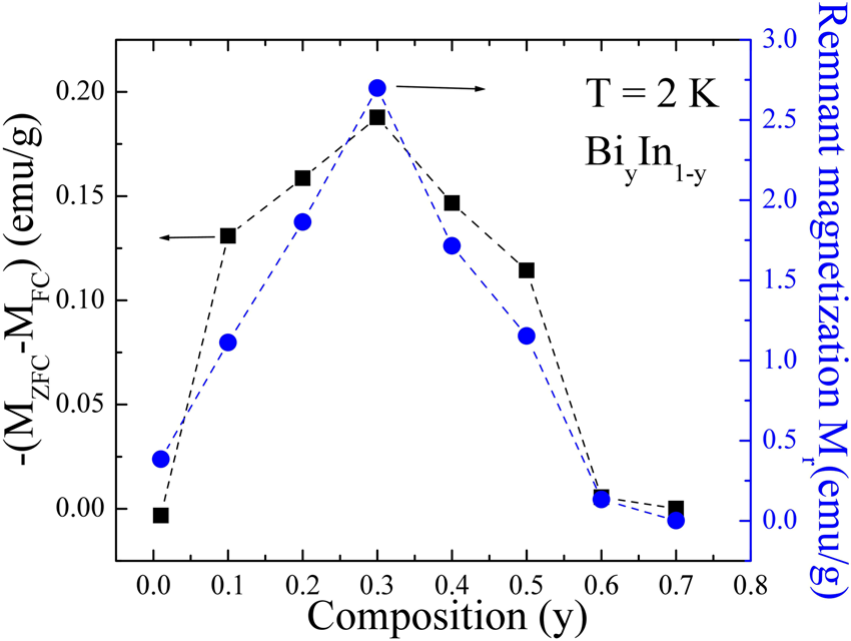
The initial composition dependence of (M_ZFC_-M_FC_) and M_r_. Plot of the absolute value of (M_ZFC_-M_FC_) (black square) and remnant magnetization M_r_ (blue circles) as a function of initial composition.

The observed overlapping and strong flux pinning from M(T) was further confirmed from the isothermal magnetization M(H_*a*_) measured at 2 K, as shown in Fig. 5(a–h). The observed pronounced reversibility for y = 0.01, *α*-In is in excellent agreement with the overlapping of FC and ZFC. The rounded shape of the M(H_*a*_) near penetration field Hp is plainly due to large local demagnetization at the corners^18^. Similar reversible behavior was also spotted for y = 0.1–0.2 and 0.6 composite samples above irreversible field H_irr_, as shown in Fig. 5(b, c) and (g). The field H_irr_ defines the transition line in the M(H_*a*_) that divides the intermediated regions into reversible and irreversible regions of flux configuration. The observed irreversibility in the intermediate state for all the samples could be because of differences in topologies upon flux entry and exit, as reported for type-I superconductors^19^. The difference in the shape of the M(H_*a*_) hysteresis of all the samples reflects how strongly the intermediate state and reversible-irreversible transition depends on grain coupling, intra-grain pinning and density of defects. For type-II superconductors, the magnetization can be defined as *m* = −*h/(1* − *N)*, where *N* is the demagnetization factor, which can vary with the direction of an external field relative to the principle axis of the superconductor, m(H_*a*_) = M(H_*a*_)/M(H_C1_) and h = (H_*a*_/H_C2_). The M(H_C1_) is the magnetization at lower critical field H_C1_, below which the superconductor retains the perfect Meissner effect, and H_C2_ is the upper critical field above which the superconductor behaves as normal conductor. The deviation of magnetization in the low field region of linearity gives the value of H_C1_^20^. The solid red lines in Fig. 5(a–h) represent the linear fit to M(H_*a*_). The goodness of linearity fit in the low field region can be represented by differential^21^, as shown in Fig. 5(i). In general, the obtained H_C1_ in M(H_*a*_) measurement suffers not only from demagnetization factor but also surface barrier effect. The type-II like hysteresis loop M(H_*a*_) then reflects the characteristic of the penetration of magnetic flux into the alloy. As can be seen in Fig. 5(e), the M(H_*a*_) loop displays an asymmetric profile in the field-increasing and -decreasing branches and a large penetration field Hp ∼ 230 Oe was obtained at T = 2 K for y = 0.4 sample. The irreversibility of the entry and exit of magnetic flux lines through the alloys surfaces shows that it is mainly the geometric edge barriers that control the movements of flux lines at the surface; a Bean–Livingston^22^ surface barrier effect has been reported in high T_C_ superconductors^23^. It should be noted that the determination of H_C1_ is of primary importance since it allows one to extract the magnetic penetration depth fundamental parameter characterizing the superconducting condensate and carrying information about the underlying pairing mechanism. A popular approach to measure H_C1_ consists of measuring the magnetization M(H_*a*_) as a function of H_*a*_ and then identify the deviation of the linear Meissner response which would correspond to the vortex penetration. One may argue that the H_C1_ values obtained from the deviation of magnetization in the low field region may not reflect the true H_C1_ but the flux entry field because of the Bean–Livingston surface barrier^22^. However, it is clear that the influence of surface barrier is important in our investigated alloys system since: (i) the magnetic hysteresis loops are asymmetric close to T_C_. (ii) Therefore, if the surface barrier should be taken into account, the true H_C1_ would be much smaller than the obtained value from the deviation of magnetization in the low field region. The upper critical field H_C2_ is defined as the field H_*a*_ at which the magnetization M(H_*a*_) shows zero value. Except for y = 0.01, *α*-In, the value of *N* is close to ~0.95(1), indicating superconducting lasting over an infinite range with H_*a*_ applied perpendicular to the surface. This accordance reveals that the coupling between the observed spherical grains from the SEM images extends over infinity. Thus, the demagnetization effect is governed by the outward appearance of the sample, not by the size and shape of each grain. In other words, as discussed before, the sample shows bulk-like superconductivity due to weakly-connected grains, even though the grain boundaries exist. Furthermore, Fig. 6 represents the calculated remnant value at zero field M_r_(0) and gives an idea about the amount of trapped flux which can be correlated with -(M_ZFC_-M_FC_) referring to the corresponding maximum value of flux excluded in the Meissner state. The highest value of M_r_(0) is for y = 0.3 composites, suggesting a larger capability to trap flux. The calculated values of H_C1_, H_C2_, penetration field Hp and demagnetization factor N, remnant magnetization M_r_, -(M_ZFC_-M_FC_), SVF and flux expulsion *f*_*ex*_ (%) at 2 K for all the samples are as tabulated in the supplementary information of Table S2.

**Figure 7:**
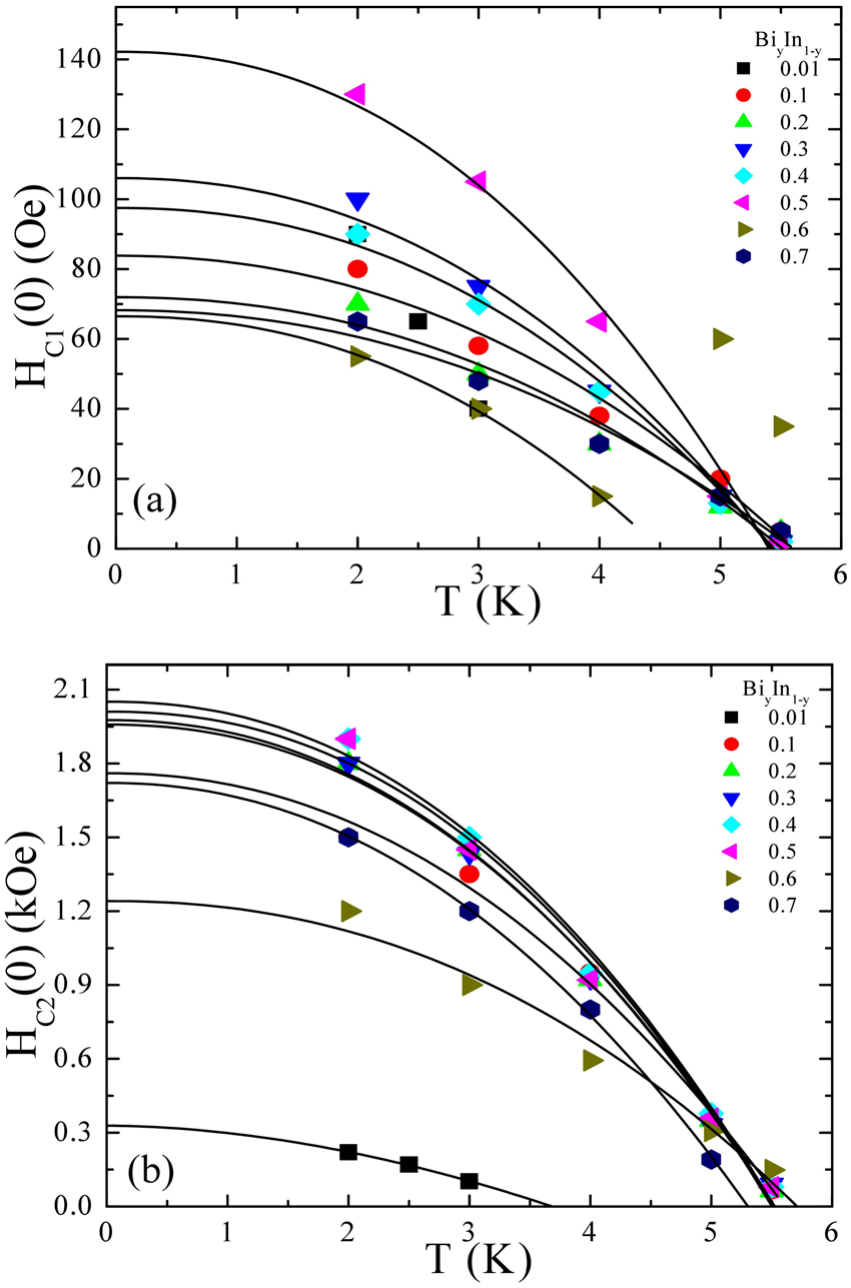
The temperature dependence of H_C1_(0) and H_C2_(0). (**a**,**b**) Lower and upper critical field as a function of temperature. Solid lines are fit to the power law mentioned in the text.

To estimate the thermal effect of the superconducting properties of a Bi_y_In_1-y_ system, M(H_*a*_) loop measurement of all the samples (y = 0.01 to 0.7) was carried out at various temperatures below the transition temperature, as can be seen in Figure S2 (see the supporting information: Figure S2). Following a similar procedure as mentioned before, the values of H_C1_ and H_C2_ were estimated at various temperatures and the observed values plotted as shown in Fig. 7(a and b). The H_C1_ and H_C2_ can be fitted with a power law, in accordance with ${H}_{C1}(T)={H}_{C1}(0)(1-\beta {(T/{T}_{C1}(0))}^{\alpha })$ and ${H}_{C2}(T)={H}_{C2}(0)(1-\delta {(T/{T}_{C1}(0))}^{\alpha })$, where *β* and *δ* are the fitting parameters with a value close to ~1(0.1), and H_C1_(0) and H_C2_(0) are the lower and upper critical field at zero temperature, respectively (see in the supplementary information of Table S2). The value of *α* represents the coupling strength obtained from the *α*-model (which will be discussed later in the text) and it was kept fixed for respective samples. The resulting fitted values of H_C1_(0) and H_C2_(0) allow us to calculate the coherence length *ξ*_*GL*_(0) and the penetration depth *λ*(0) using a Ginzburg-Landau (GL) formulation^24^, ${\xi }_{GL}(0)=\sqrt{{{\rm{\Phi }}}_{0}/2\pi {H}_{C2}(0)}\,$and $\lambda (0)=\sqrt{{{\rm{\Phi }}}_{0}/2\pi {H}_{C1}(0)}$, where ${{\rm{\Phi }}}_{o}=\frac{{\rm{h}}}{2{\rm{e}}}=2.0678\times {10}^{9}\,{\rm{O}}{\rm{e}}\,{A}^{2}$ is the quantum flux^25^, which yields minimum *ξ*_*GL*_(0) ~ 40.1 nm (y = 0.4) and maximum *λ*(0) ~ 220 nm (y = 0.7) at 0 K. Using the relation ${\rm{\kappa }}(0)={\rm{\lambda }}(0)/{\xi }_{GL}(0)$ we calculated the value κ(0), which varied from 1.6 (y = 0.01) to 5.0 (y = 0.7), indicating type II superconducting behavior. In addition, the thermodynamic critical field, ${H}_{TC}(0)\approx \sqrt{{H}_{C1}(0){H}_{C2}(0)}\,$was calculated, showing a maximum value of ~534 Oe for the In_2_Bi superconductor (y = 0.5). The estimated superconducting parameters for all the BiIn samples are as tabulated in the supplementary information of Table S3.

### Applied magnetic field dependency of ZFC and FC Magnetization

**Figure 8:**
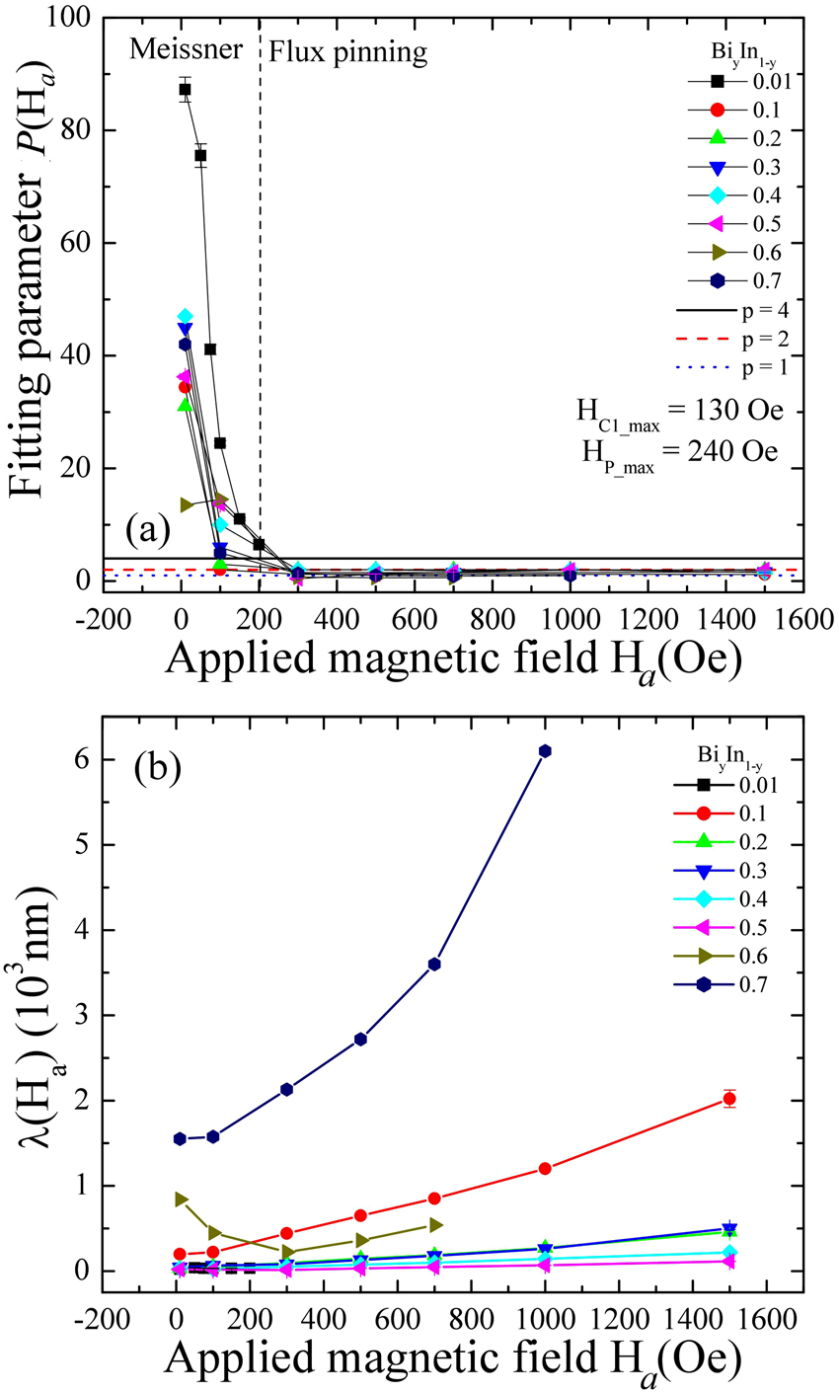
The applied magnetic field dependence of *P*(H_*a*_) and λ(H_*a*_). Applied magnetic field dependence of the fitted values of (**a**) *P*(H_*a*_) and (**b**) London penetration depth λ(H_*a*_), respectively.

**Figure 9:**
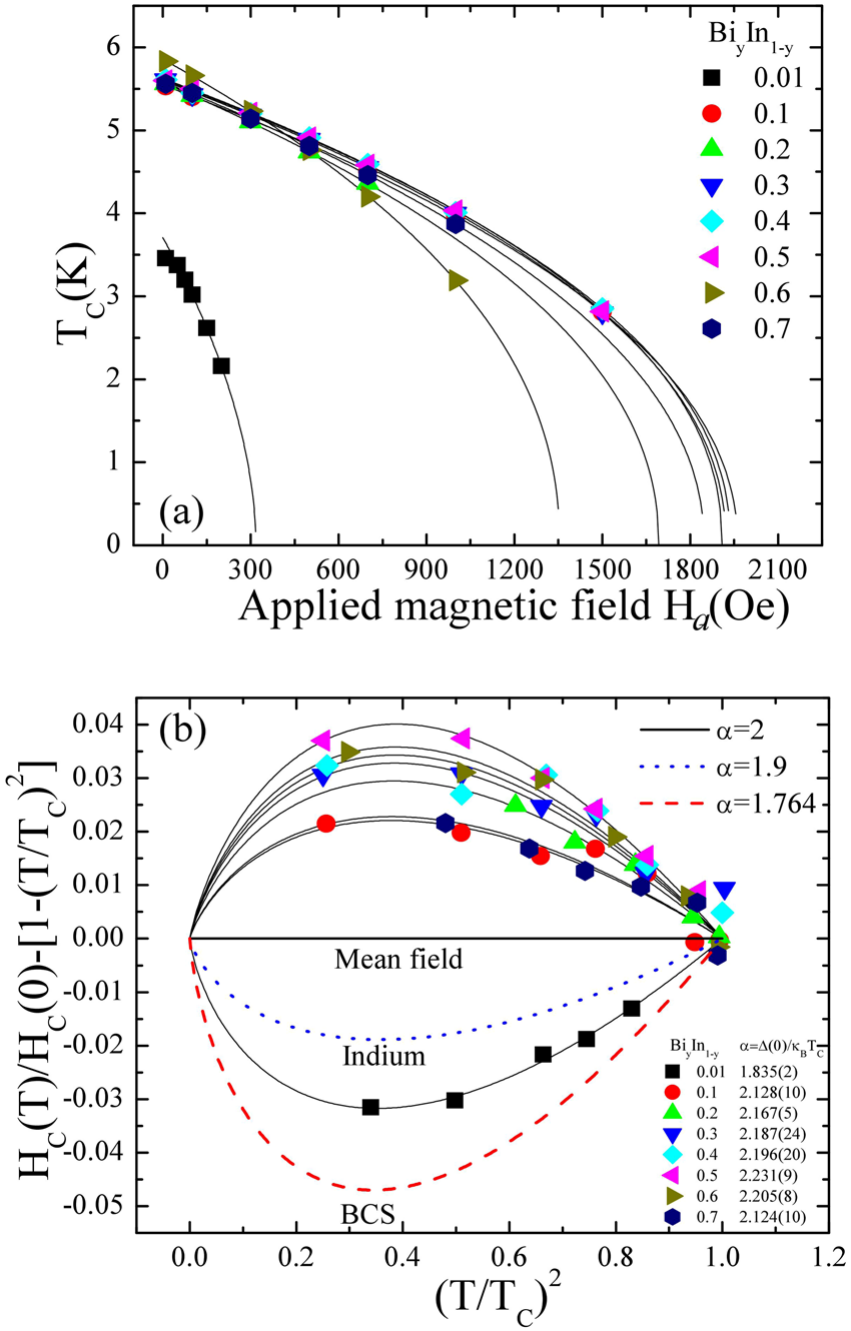
The applied magnetic field dependence of T_C_(H_*a*_) and coupling strength analysis. (**a**) The effect of applied field H_*a*_ on the transition temperature T_C_ of BiIn samples. The solid line is fitted to the expression given in the text. (**b**) Deviation of H_C_(T)/H_C_(0) from the parabolic dependency of 1 − (T/T_C_)^2^ fitted to the expression ${H}_{C}(T)/{H}_{C}(0)=[1-{(T/{T}_{C})}^{\alpha }]$, where α represents the coupling strength of the superconductor.

The cause behind an enhanced transition temperature of In_2_Bi and In_5_Bi_3_ has not been clearly identified yet. There are a few possibilities, such as internal strain or short range disorder, as reported for an InSn system, or the enhanced electron-phonon coupling mediated by low lying phonons, as observed for a BiPb system. The effect due to a short range disorder can be avoided, as the calculated mean free path at 2 K is a multiple of lattice constants. From GL theory, the carrier mean free path, $\ell$, at low temperature T = 2 K can be estimated using

2 $$\frac{1}{\ell }=\frac{{\xi }_{o}}{0.882}[\frac{0.546}{{\xi }_{GL}^{2}(0)(1-T/{T}_{C})}-\frac{1}{{\xi }^{2}(0)}],$$

where *ξ*(0) = 3640 Å is the BCS coherence length of bulk In^26^. To estimate the superconducting coupling strength, we carried out ZFC measurement at various external applied field H_*a*_, as shown in Figure S3 (see the supporting information: Figure S3). The Meissner diamagnetic screening signal appears to shift consistently to a lower temperature as H_*a*_ is increased. The relatively sharp distribution of the transition temperature from the y = 0.01 sample, as compared to other superconductors, signals that *α*-In remains in the Meissner state below 150 Oe. The results are in good agreement with the estimated lower critical field H_C1_(0) = 134(3) Oe for α-In and the maximum H_C1_(0) = 106(5) Oe for the remaining samples. A broad distribution of transition temperatures, extending to the lowest available temperature at field H_*a*_ > 100 Oe, was observed for sample y = 0.1 to 0.7. Schuck *et al*. from single crystal KOs_2_O_6_ and Rogacki *et al*.^27, 28^ from single crystal RbOs_2_O_6_ reported the observation of similar broad distributions of transition temperatures to what was observed in the InBi system. However, they correlated the broad distribution of transition temperatures with weak-pinning behavior, which is contradictory to a BiIn system. The observed broad distribution could be the sign of a weakly-linked granular superconductor, as these BiIn superconductors include both weak and strong-pinned superconductors. The fitted value of *P* to the ZFC of all the superconducting samples within the Meissner state is higher than 4, whereas in the flux pinning state it lies between 1 to 2. Therefore, values of *P* can help in defining the state of the linked intergranular superconducting system. The fitted values of *P*(*H*_*a*_) and the London penetration depth λ(H_*a*_) are as plotted in Fig. 8(a and b), respectively. Within a Meissner state λ(H_*a*_) remains constant or shows slightly decreasing behavior with an increase of H_*a*_ (≤H_C1_(0)). The reduction of the diamagnetic screening in the flux pinning state is known to be caused by the increase of λ with H_*a*_, which in turn reduces the SVF, hence the shielding capability of a superconductor^29^. Furthermore, the suppression of critical temperature with an increase of field H_*a*_ can be well described using ${T}_{C}({H}_{a})={T}_{C}(0){[1-{H}_{a}/{H}_{c}(0)]}^{\gamma }$, where T_C_(0) is the zero field critical temperature, H_C_(0) is the zero temperature critical field, and *γ* is a fitting parameter, as shown in Fig. 9(a). All the fitting parameters are as tabulated in the supplementary information of Table S4. The fitted value of H_C_(0) for each sample is close to the H_C2_(0) and the maximum value is ~6.5 times that of the pure Indium phase. Padamsee *et al*.^30^ introduced the so-called *α*-model, by which the relative coupling strength, α = Δ(0)/*k*_*B*_ *T*_*C*_ (where, Δ(0) is the superconducting energy gap at 0 K and k_B_ is the Boltzmann constant) of a superconducting system can be revealed in the deviation of H_C_(T)/H_C_(0) from the parabolic dependency of 1 − (*T*/*T*_*C*_)^2^. It is known that weak-coupled systems yield negative deviations, while strong-coupled systems yield positive deviations^31^. The observed negative deviation for *α*-In (y = 0.01) and the positive deviation for the remaining BiIn superconductor (y = 0.1 to 0.7) can be fitted by using $\frac{{H}_{C}(T)}{{H}_{C}(0)}=[1-{(\frac{T}{{T}_{C}})}^{\alpha }]$, as shown in Fig. 9(b). The solid line represents the fitted curve, and the fitted values of *α* are as shown in the supplementary information of Table S4. A minimum of *α* = 1.835(2) (close to weak-coupled bulk In, 1.9) from y = 0.01 and a maximum of 2.231(9) (larger than strong-coupled bulk Pb) from y = 0.5, indicate the pure In_2_Bi phase is larger than BCS prediction of 1.764. The fitted values of the coupling strength for a In_2_Bi superconductor could be mediated by low lying phonons resulting in an enhanced T_C_. The average value of logarithmic phonon energy ω_ln_ and the dimensionless electron-phonon coupling constant λ_ep_ for a sample of BiIn can be calculated from the Eliashberg theory-based Allen-Dynes formulation^2^. The corrections of the BCS values by strong electron-phonon interactions have been deduced in the following approximate analytic formulas that link ω_ln_/T_C_ to experimental thermodynamic quantities:

3 $${\rm{\alpha }}=\frac{{\rm{\Delta }}(0)}{{k}_{B}{T}_{C}(0)}=1.764[1+12.5{({T}_{C}(0)/{\omega }_{ln})}^{2}\times ln({\omega }_{ln}/2{T}_{C}(0))],$$

**Figure 10:**
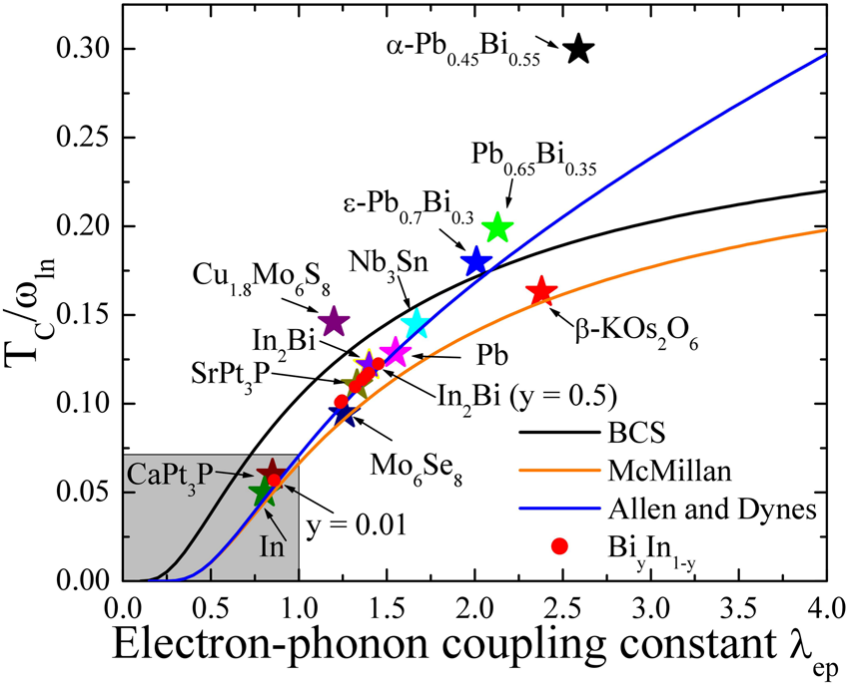
The dependence of T_C_/ω_ln_ on λ_ep_. The plot of T_C_/ω_ln_ as a function of the electron-phonon coupling constant λ_ep_ of BiIn (red circles) calculated from the Allen and Dynes modified McMillan formula (blue solid line). The black and orange colored solid lines represent calculated T_C_/ω_ln_ using BCS and McMillan’s formula. For comparison, the different phonon mediated superconductors (stars) are also shown.

The calculated values of ω_ln_ are as shown in Table S4, and we see that a minimum of 45.92 K was calculated for the pure In_2_Bi sample (y = 0.5). Next, the dimensionless λ_ep_ can be estimated from McMillan’s formulation^32^. However, as can be seen from Fig. 10, the McMillan formulation is useful only for intermediate coupled superconductors, λ_ep_ < 1. As shown in Fig. 10, most of the phonon mediated superconductors can be well described using Allen and Dynes’ modified McMillan formula^2^,

4 $$\frac{{T}_{C}}{{\omega }_{ln}}=\frac{{f}_{1}{f}_{2}}{1.2}exp[\frac{-1.04(1+{\lambda }_{ep})}{{\lambda }_{ep}-{\mu }^{\ast }(1+0.62{\lambda }_{ep})}].$$

where ${f}_{1}={[1+{(\frac{{\lambda }_{ep}}{{{\rm{\Lambda }}}_{1}})}^{3/2}]}^{1/3}$is a strong coupling correction function and ${f}_{2}=1+(\frac{{\varpi }_{2}}{{\omega }_{ln}}-1){\lambda }_{ep}^{2}/({\lambda }_{ep}^{2}+{{\rm{\Lambda }}}_{2}^{2})$ is a shape correction function. Where ${{\rm{\Lambda }}}_{1}=2.46(1+3.8{\mu }^{\ast })$, ${{\rm{\Lambda }}}_{2}=1.82(1+6.3{\mu }^{\ast })(\frac{{\varpi }_{2}}{{\omega }_{ln}})$ and ${\varpi }_{2}=\sqrt{ < {\omega }_{ln}^{2} > }$ is the square root average logarithm of the phonon frequency introduced by Allen and Dynes, and *μ* ^*^ is the Coulomb pseudo potential. The *μ* ^*^ describes the Coulomb pseudopotential to represent the repulsive part of the pairing interaction, and to calculate λ_ep_ we set *μ* ^*^ = 0.11 and ${\varpi }_{2}=1.3{\omega }_{ln}$. The plot of T_C_/ω_ln_ vs. λ_ep_ of the BiIn system (red filled circle) along with different phonon mediated superconductors is as shown in Fig. 10. The blue solid line represents the calculated *T*_*C*_/*ω*_*ln*_ using Allen and Dyne’s formula. For comparison, we have also shown the results from the BCS and McMillan formulation (black and orange solid lines, respectively). From the above Fig. 10, for λ_ep_ < 1, for the Allen and Dyne formulation reduced to McMillan formulation^32^ and for λ_ep_ < 0.2, both the Allen-Dyne and McMillan formulations are reduced to the BCS prediction^33, 34^. The calculated value of λ_ep_ = 1.453, ω_ln_ = 45.92 K for BiIn is in good agreement with the earlier reported value of λ_ep_ = 1.40, ω_ln_ = 46 K^2^. However, using McMillan’s formula with *μ* ^*^ = 0.11 and assuming ω_ln_ to be same as a Debye frequency, we found that the ratio T_C_/ω_ln_ for In_2_Bi is reproduced with relatively high λ_ep_ ~1.68, which contradicts previous findings. In order to match the experimental value of T_C_ the required λ would be 1.2–1.4 (with reasonable Coloumb pseudo potential μ* of 0.06–0.09), reflecting the uncertainty in the estimation of Coloumb pseudo potential. It is known that disorder/substitution can affect the Coulomb pseudo potential in superconductor^35^. Hence we could infer that the local inhomogeneity introduced by disorder/substitution in bimetallic BiIn alloys may play nontrivial role in the influence of Coulomb pseudo potential. Indeed, the intimate relationship between local inhomogeneity and superconductivity has been extensively studied in the context of high T_C_ superconductors, and the possible enhancement of superconductivity by local inhomogeneities has been discussed recently by Martin *et al*.^36^ in the weak coupling BCS regime.

From Fig. 10, we can conclude that enhanced T_C_ is the combined effect of phonon energy and the electron-phonon coupling. For example, ε-Pb_0.7_Bi_0.3_ (λ_ep_ ~ 2.01, ω_ln_ = 47 K) and Pb_0.8_Bi_0.2_ (λ_ep_ ~ 1.88, ω_ln_ = 46 K) have the same phonon energy as In_2_Bi, but higher values of λ_ep_, resulting in enhanced Tc of 8.45 K and 7.95 K, respectively^2^. Similarly, Mo_6_Se_8_ (λ_ep_ = 1.27, ω_ln_ = 70 K) and SrPt_3_P (λ_ep_ = 1.33, ω_ln_ = 77 K) which overlap InBi superconductors, as shown in the figure, also show enhanced T_C_ of 8.5 and 6.34 K, respectively^3, 4^. Since, λ_ep_ for these two superconductors is lower than In_2_Bi, but with high value of phonon energy. Moreover, there are a few superconductors, as shown in the figure, which are beyond Allen and Dynes’ prediction, and therefore more theoretical work needs to be carried out. It is known that disorder, substitution in weak coupling superconductivity could enhance the electron-phonon coupling constant. A simplified physical model of electron-phonon coupling has been developed by Gao *et al*.^37^ to allow heat transfer from phonons to electrons, and applied to study defect or disorder as a function of the strength of electron-phonon coupling. The number of point defects produced in the primary damage state increases with increasing strength of electron-phonon coupling, implying the defect or disorder/substitution plays a role in the increasing strength of electron-phonon coupling constant. As reported in Cu_*x*_Bi_2_Se_3_F superconductor by Zhang *et al*.^35^, first principle calculation was introduced to investigate the role of electron-phonon coupling for the superconducting pairing in the prime candidate Cu_*x*_Bi_2_Se_3_. In their comprehensive analysis reveal that electron-phonon scattering process in this system is dominated by zone center and boundary optical modes, with coexistence of phonon stiffening and softening, suggesting that superconductivity may not only come from pure electron-phonon coupling but also disorder effect. In the present work, we also cannot ignore the possible enhancement of electron-phonon coupling by local inhomogeneity introduced by doping defect or disorder/substitution in bimetallic BiIn alloys. From the above finding, it’s clear that the observed enhanced T_C_ for a Bi_y_In_1-y_ system (i.e. In_2_Bi and In_5_Bi_3_) is the effect of strong electron-phonon coupling to low-lying phonons. The effect of low lying phonons can be further proved by carrying out electron-tunneling spectroscopy measurements.

## Discussion

The strong-coupling superconducting behavior mediated by low lying phonons has been reported to be the cause for enhanced T_C_ in Bi_y_In_1-y_ superconductors. The maximum strong-coupling superconducting strength, α = 2.231(9), phonon energy, ω_ln_ = 45.9 K, electron-phonon coupling constant, λ_ep_ = 1.453, and critical field, H_C_(0) = 1909(3) Oe has been estimated from pure In_2_Bi (y = 0.5) samples using Allen and Dynes’ formulation. The different superconducting parameters estimated using GL formulation show Bi_y_In_1-y_ is a type-II superconductor with GL constant, 1.6 ≤ k(0) ≤ 5 for 0.01 ≤ y ≤ 0.7. The room temperature SR-XRD refinement carried out by using weighted analysis and superconducting measurement reveals the formation of both pure α-In (y = 0.01), In_2_Bi (y = 0.5) and composite α-In + In_2_Bi (y = 0.1–0.4), In_2_Bi + In_5_Bi_3_ + InBi (y = 0.6–0.7) samples, respectively. The ZFC-FC temperature dependent measurement revealed two-gap superconductivity in y = 0.1–0.4 (*α*-In + In_2_Bi) and 0.6–0.7 (In_2_Bi + In_5_Bi_3_) and a single gap in y = 0.01(*α*-In) and y = 0.5 (In_2_Bi) superconductors, respectively. The highest transition temperature was determined to be T_C1_(10 Oe) = 5.85 K and T_C2_ (10 Oe) = 4.27 K (y = 0.6) when fitted to the London equation with free fitting parameter P. The value of P gives an idea about the distribution of transition temperatures and can help in naming the Meissner/flux pinning state. The SVF estimated from ZFC revealed bulk-like superconductivity for all the superconducting samples. The high and low values of flux expulsion calculated from FC revealed that y = 0.01, 0.6–0.7 are strong-linked, whereas y = 0.1 to 0.5 are relatively weak-linked intergranular superconductors. The estimated demagnetization factor *N* ~ 0.95(1) (y = 0.1 to 0.7) from field dependent magnetization measurement signals that linkage within the grains, as observed from SEM images, extends over infinity. We argue that the observed broad distribution of transition temperatures in the flux pinning state is the special feature of strong/weak-linked granular superconductors. Finally, in this report, we have demonstrated the potentiality of superconducting measurement over the SR-XRD for identifying very small volume fractions of a superconducting phase.

## Methods

Bi_y_In_1-y_ (0.01 ≤ y ≤ 0.7) superconductors were prepared by using physical solid state reaction. This tuning of the Bi concentration alters the crystal structure, and thereby changes its superconducting properties. Surface morphological analysis and atomic percentage calculation of all the samples were performed by field-emission scanning electron microscopy (FE-SEM) using a JEOL JSM-6500F microscope (JEOL Ltd., Tokyo, Japan). Energy dispersive spectroscopy (EDS; Inca x-sight model 7557, Oxford Instruments, Abingdon, Oxfordshire, U.K.) was utilized to estimate the atomic percentages of the constituent elements. Energy-dispersive spectroscopy (EDS) is a useful technique for estimating the atomic percentages of constituent elements in the samples.

## Electronic supplementary material

Supplementary information
