# Supplementary material for: Routes to probe Bismuth induced strong-coupling superconductivity in bimetallic BiIn alloys
Source: Sci Rep. 2017 Aug 25;7:9442. doi: 10.1038/s41598-017-09831-9 (PMC5572680; doi:10.1038/s41598-017-09831-9)
Supplement: Supplementary file 1 — Supplementary information [file 41598_2017_9831_MOESM1_ESM.pdf]

Supplementary information

Routes to probe Bismuth induced strong-coupling superconductivity in bimetallic BiIn alloys

Ashish Chhaganlal Gandhi & Sheng Yun Wu*

Department of Physics, National Dong Hwa University, Hualien 97401, Taiwan

Figure S1 (a) Representation of state of alloy depending on initial composition y and the corresponding crystal structure of In, In_2Bi, In_5Bi_3, InBi and Bi. (b)-(d) Represents refined lattice parameters for In-solid solution, In_2Bi, In_5Bi_3 and InBi, respectively.

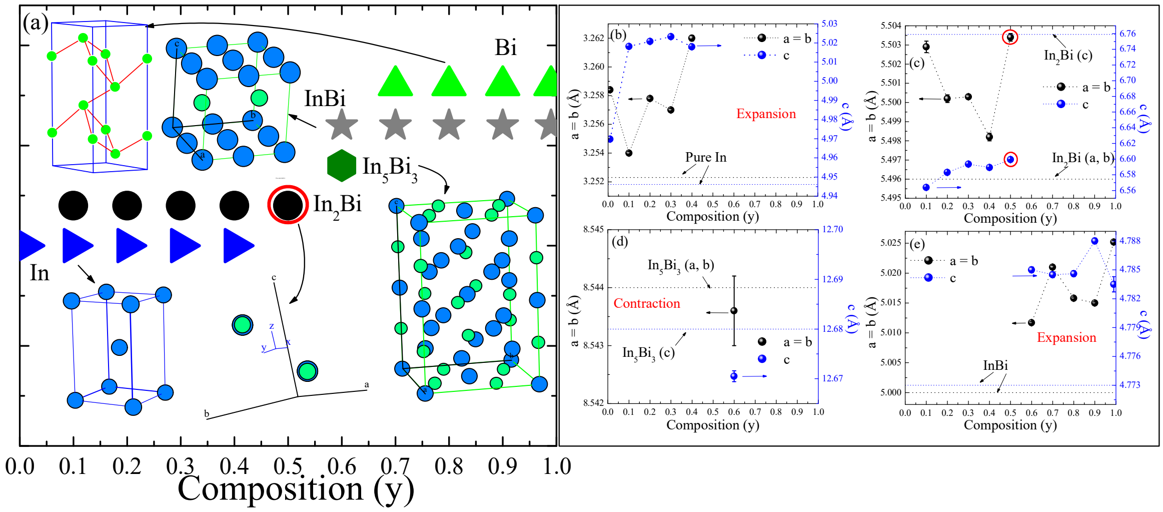

Figure S2 (a)-(h) Applied magnetic field dependence of magnetization measured at various temperatures.

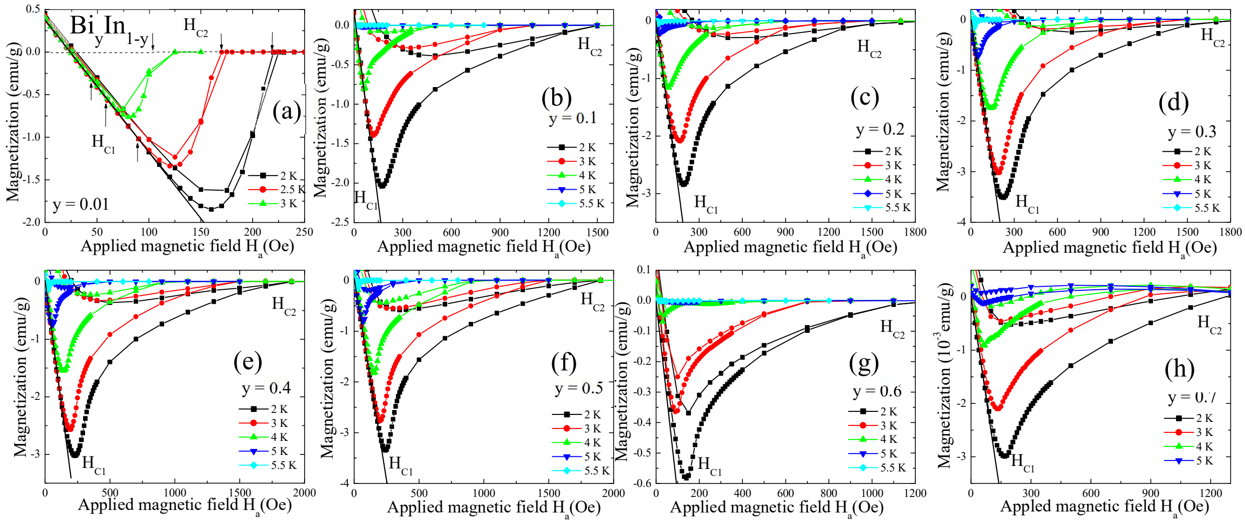

Figure S3 (a)-(h) Temperature dependence of magnetization measured at various applied magnetic fields.

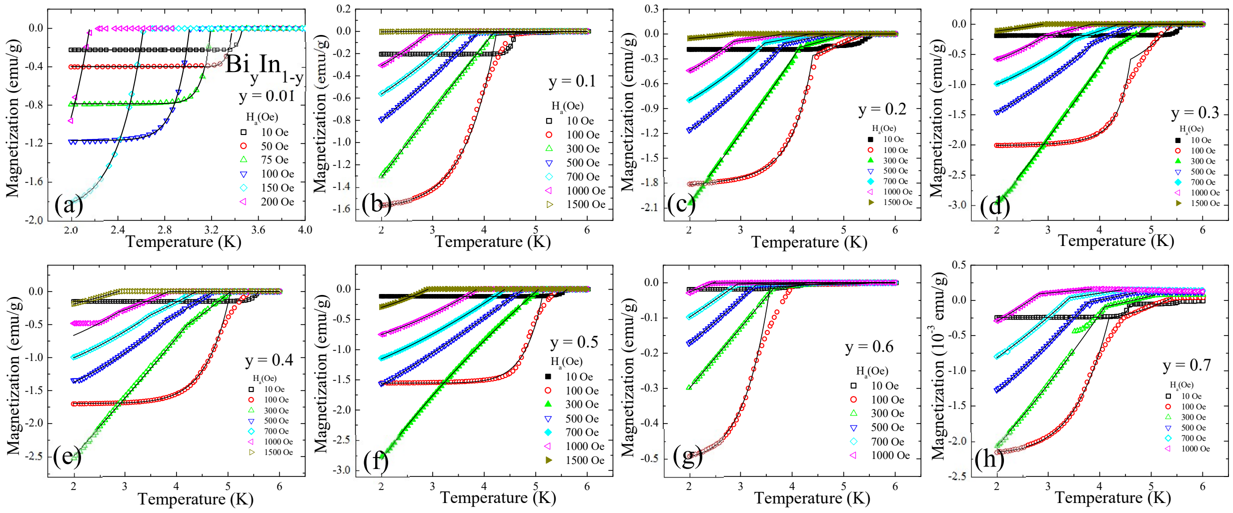

Figure S4 Allen and Dyne formulation simulation for Electron-phonon coupling constant vs T_C at various Coulomb pseudo potential, from which we can estimate the lower bound of λ to be ~1.453 to match the experimental T_C of 5.624 K (as μ^*=0.11).

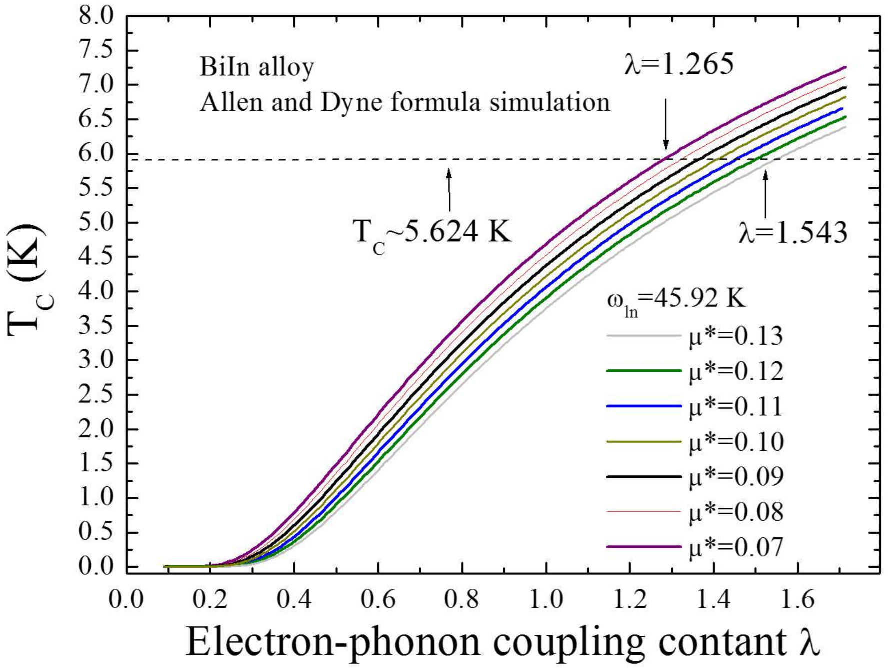

Table S1 Summary of the fitting parameters obtained from the Rietveld refinement of the XRD spectra (weight fraction, $a = b$, c, deviation, χ^2, R_p, R_{wp}).

Material	Space group	Lattice parameters				Atomic positions			χ^2	*c/a*
		a = *b* (Å)	*c* (Å)	α = β (deg.)	γ (deg.)	x	y	z		
Bi	R-3m (No. 166)	4.533	11.797	90	120	0	0	0.2327(2)		2.6025
In	I4/mmm (No. 139)	3.2523	4.9461	90	90	0	0	0		1.5208
In_2Bi (y = 0.5)	P63/mmc (No. 194)	5.5034(22)	6.5994(27)	90	120	Bi-1/3	2/3	3/4	0.478	1.1992
						In-2/3	1/3	1/4		
						Bi-0.343	0.843	0		
In_5Bi_3 (y = 0.6)	I4/mcm (No. 140)	8.5436(6)	12.6705(11)	90	90	Bi-0	0	1/4		1.4830
						In-0.144	0.644	0.168		
						In-0	0	0		
									1.076	
						Bi-1/4	1/4	0.393		
InBi (y = 0.6)	P4/nmm (No.129)	5.0117(2)	4.7850(3)	90	90	Bi-3/4	3/4	-0.393		0.9548
						In-0	0	0		
						In-1/2	1/2	0		

Table S2 Summary of the calculated values of H_{C1}, H_{C2}, penetration field H_p and demagnetization factor *N*, remnant magnetization Mr, –(M_{ZFC}-M_{FC}), SVF and flux expulsion f_{ex} (%) at 2 K for all the samples.

Bi_yIn_{y-1}	H_{c1} (Oe)	H_p (Oe)	H_{c2} (Oe)	N	$M_r(0)$	-(M_{ZFC}-M_{FC})	SVF(%) ZFC	f_{ex} (%)
0.01	90	160	220	0.7014	0.3844	-0.0033	179	101
0.1	80	170	1500	0.9539	1.1119	0.1309	163	36
0.2	70	190	1800	0.9614	1.8634	0.1587	148	15
0.3	100	230	1900	0.9479	2.6993	0.1878	150	0.5
0.4	90	230	1900	0.9534	1.7154	0.1467	118	1.3
0.5	130	240	1900	0.9326	1.1527	0.1144	94	2.7
0.6	55	140	1200	0.9671	0.1329	0.0055	15	70
0.7	65	170	1500	0.9604	0.0011	0.0001	0.2	58

Table S3 Summary of fitting parameters of $H_{TC}(0)$, $\xi(0)$, and $\lambda(0)$ to the FC and ZFC temperature dependent magnetization curve, respectively.

Bi_yIn_{1-y}	$H_{TC}(0)$ (Oe)	$\xi(0)$ (Å)	$\lambda(0)$ (Å)
0.01	210	1000	1568
0.1	385	432	1980
0.2	377	408	2138
0.3	456	410	1762
0.4	446	401	1842
0.5	534	405	1523
0.6	288	515	2217
0.7	342	437	2200

Table S4 Summary of fitting parameters of κ, ℓ, $T_c(0)$, $H_C(0)$, γ, α, ω_{ln} and λ_{ep} to the applied magnetic field dependent magnetization curve, respectively.

Bi_yIn_{1-y}	κ	ℓ(Å)	$T_C(0)$ (K)	$H_c(0)$ (Oe)	γ	α	ω_{ln} (K)	λ_{ep}
0.01	1.6	240	3.887(19)	318(4)	0.545(13)	1.835(2)	68.45	0.861
0.1	4.6	65	5.545(7)	1962(20)	0.470(10)	2.128(10)	54.57	1.245
0.2	5.2	45	5.576(4)	1693(78)	0.462(28)	2.167(5)	50.91	1.321
0.3	4.3	45	5.598(15)	1922(38)	0.457(19)	2.187(24)	49.31	1.361
0.4	4.6	45	5.611(14)	1937(38)	0.456(19)	2.196(20)	48.64	1.38
0.5	3.8	46	5.624(5)	1909(13)	0.448(7)	2.231(9)	45.92	1.453
0.6	4.3	81	5.856(7)	1355(15)	0.454(10)	2.205(8)	49.97	1.399
0.7	5.0	61	5.584(7)	1848(67)	0.471(25)	2.124(10)	55.45	1.237
